# Supplementary material for: Soluble Collectin 11 (CL-11) Acts as an Immunosuppressive Molecule Potentially Used by Stem Cell-Derived Retinal Epithelial Cells to Modulate T Cell Response
Source: Cells. 2023 Jul 7;12(13):1805. doi: 10.3390/cells12131805 (PMC10341155; doi:10.3390/cells12131805)
Supplement: Supplementary file 1 [file cells-12-01805-s001.zip › cells-2421777-supplementary.pdf]

Supplementary Materials

Soluble Collectin 11 (CL-11) acts as an immunosuppressive molecule potentially used by stem cell derived retinal epithelial cells to modulate T cell response

Giorgia Fanelli[1*], Marco Romano[1], Giovanna Lombardi[1] and Steven H. Sacks[1]

[1] Peter Gorer Department of Immunobiology, School of Immunology and Microbial Sciences, King's College London, SE1 9RT, UK.

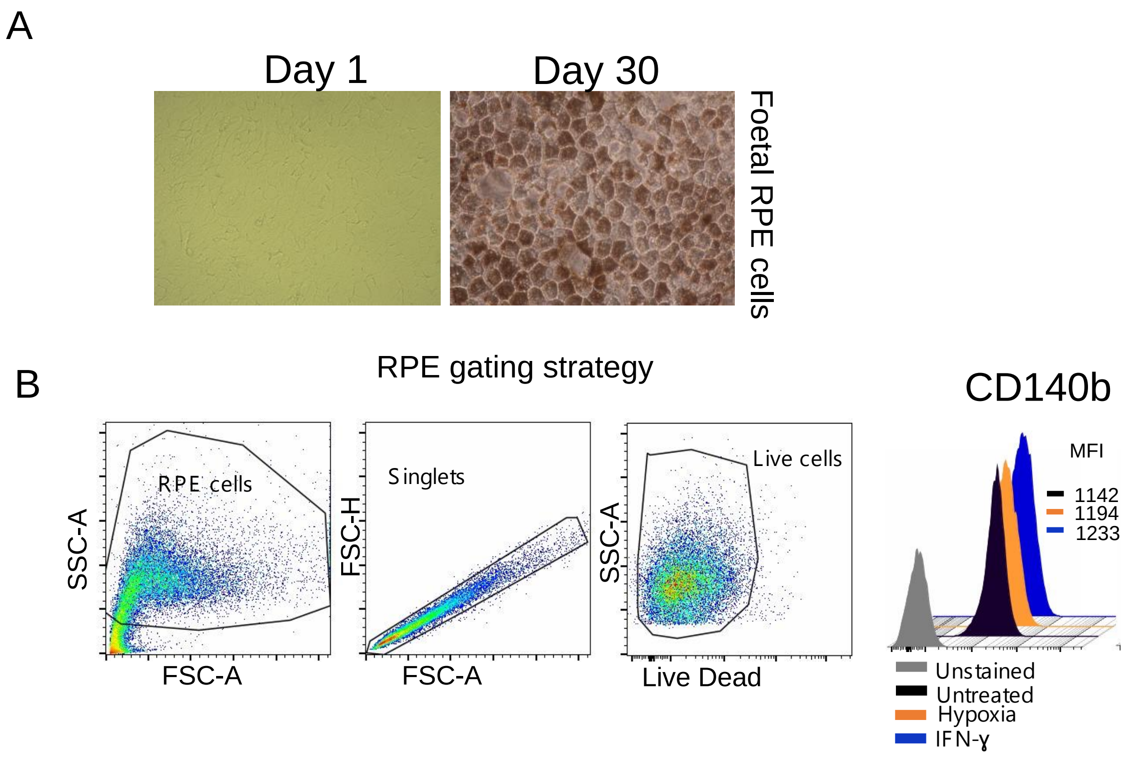

Figure S1. fRPE cell *in vitro* differentiation. (**A**) Phase contrast microscopy showing key morphological changes prior to differentiation (day 1). A cobblestone appearance and homogeneous pigmentation was visible in the RPE cell population by day 30 of cell culture. (**B**) Representative flow cytometry plots showing the gating strategy of fRPE cells used in this study and a representative flow cytometry histograms showing surface expression of CD140b, on fRPE cells treated for 24h under hypoxic conditions or for 3 days with 500 IU of hrIFNg.

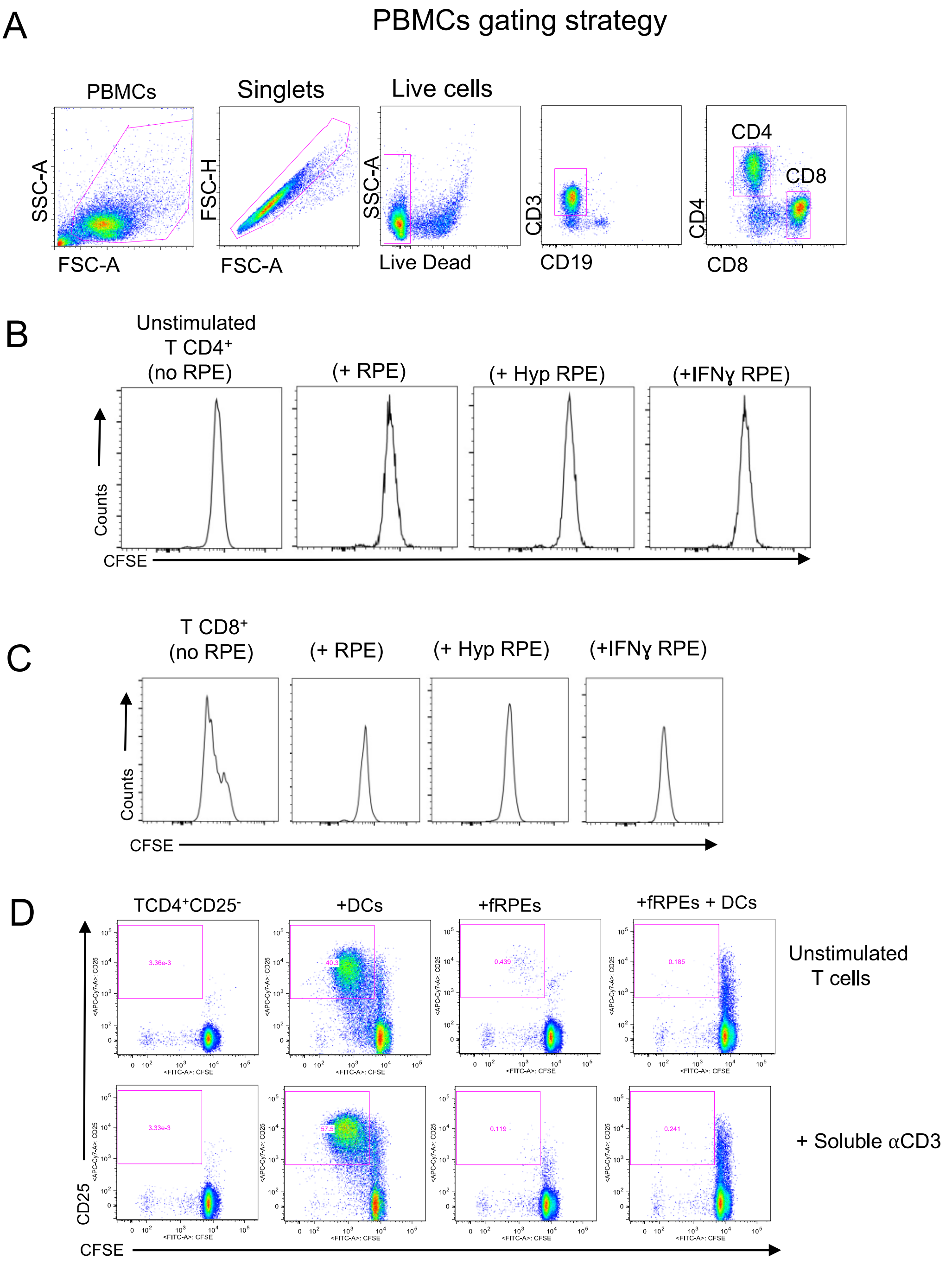

Figure S2. PBMCs gating strategy and co-culture assay with fRPE cells. (**A**) PBMCs gating strategy to assess cell proliferation of CD4^{+} and CD8^{+} T cell subsets. (**B**) Representative histogram showing CFSE dilution of gated CD4^{+} T cells cultured for 4 days alone or in the presence of fRPE cells pre-treated as indicated. (**C**) Representative histogram showing CFSE dilution of gated CD8^{+} T cells activated with soluble αCD3

(0.5 µg/mL) and cultured for 4 days alone or in the presence of fRPE cells pre-treated as indicated. **(D)** Representative plots showing proliferation (CFSE dilution) and CD25 expression of CD4^{+}CD25^{-} T cells unstimulated (first row) or stimulated with soluble αCD3 (0.5 µg/mL) (second row) in the presence or not of allogeneic dendritic cells (DCs), fRPE cells or fRPE cells and DCs.

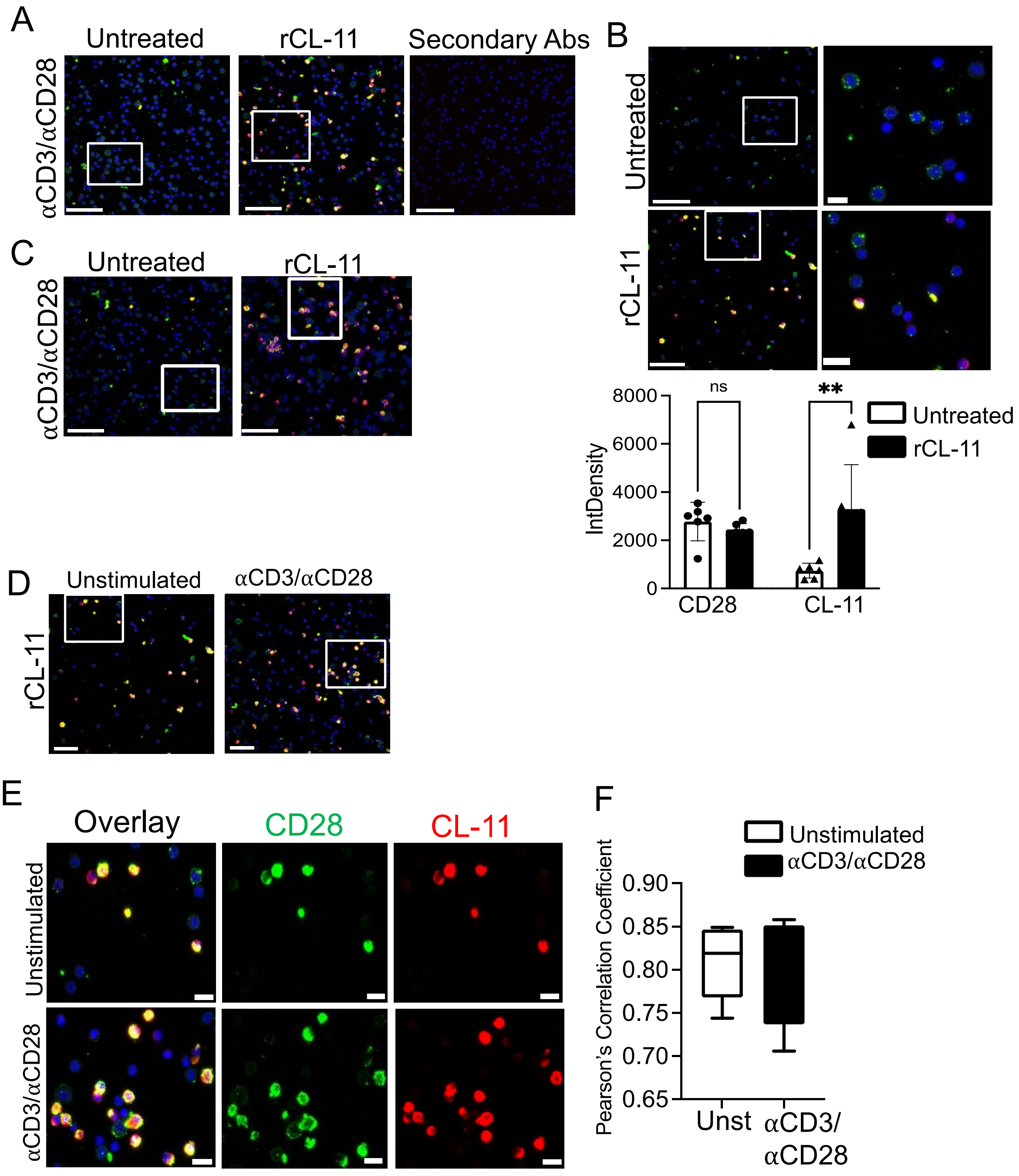

Figure S3. Uncropped confocal images and CD28 and CL-11 correlation. (**A**) Representative uncropped confocal images with the selected areas (white rectangle) shown in the main figure of the manuscript (Figure 3A). Cell stained with goat anti-mouse Alexa fluor 488 and goat anti-rabbit Alexa Fluor 546 secondary antibodies alone were used as a control. CD28 (green), CL-11 (red) and Nuclei (blue) are shown. Scale bars represent 50 μM. (**B**) Representative confocal images showing unstimulated cells pre-incubated for 1h at 37°C with 20μg/mL rCL-11 or buffer solution. Uncropped images (scale bars represent 50 μM) are on the left with the selected areas (white rectangle) shown in the right panels (scale bars represent 25 μM). Quantification of integrated density for CD28 and CL-11 fluorescence using ImageJ software. Data are expressed as mean ± SEM. 8 cropped images per condition of a minimum of 20 cells are pooled from three different healthy. $^{**}P<0.01$ was considered significant using Two-way ANOVA followed by Šídák multiple comparison (Bottom panel). (**C**) Representative uncropped confocal images with the selected areas (white rectangle) shown in the main figure of the manuscript (Figure 3E). Scale bars represent 50 μM. (**D**) Representative uncropped confocal images with the selected areas (white rectangle) shown in Supplementary Figure 3E. Scale bars represent 50 μM. (**E**) Representative cropped images showing CD28 (green), CL-11 (red) and Nuclei (blue) overlay and CD28 (green) and CL-11 (red) single channels on $CD4^{+}CD25^{-}$ T cells activated or not with αCD3/αCD28 for 48 hours and pre-incubated for 1h at RT with 20μg/mL rCL-11. Scale bars represent 10 μM. (**F**) Statistical analysis for colocalization events of CL-11 and CD28 with Pearson's Correlation Coefficient method (ImageJ software). Data are represented using boxplots indicating the min and max and median.

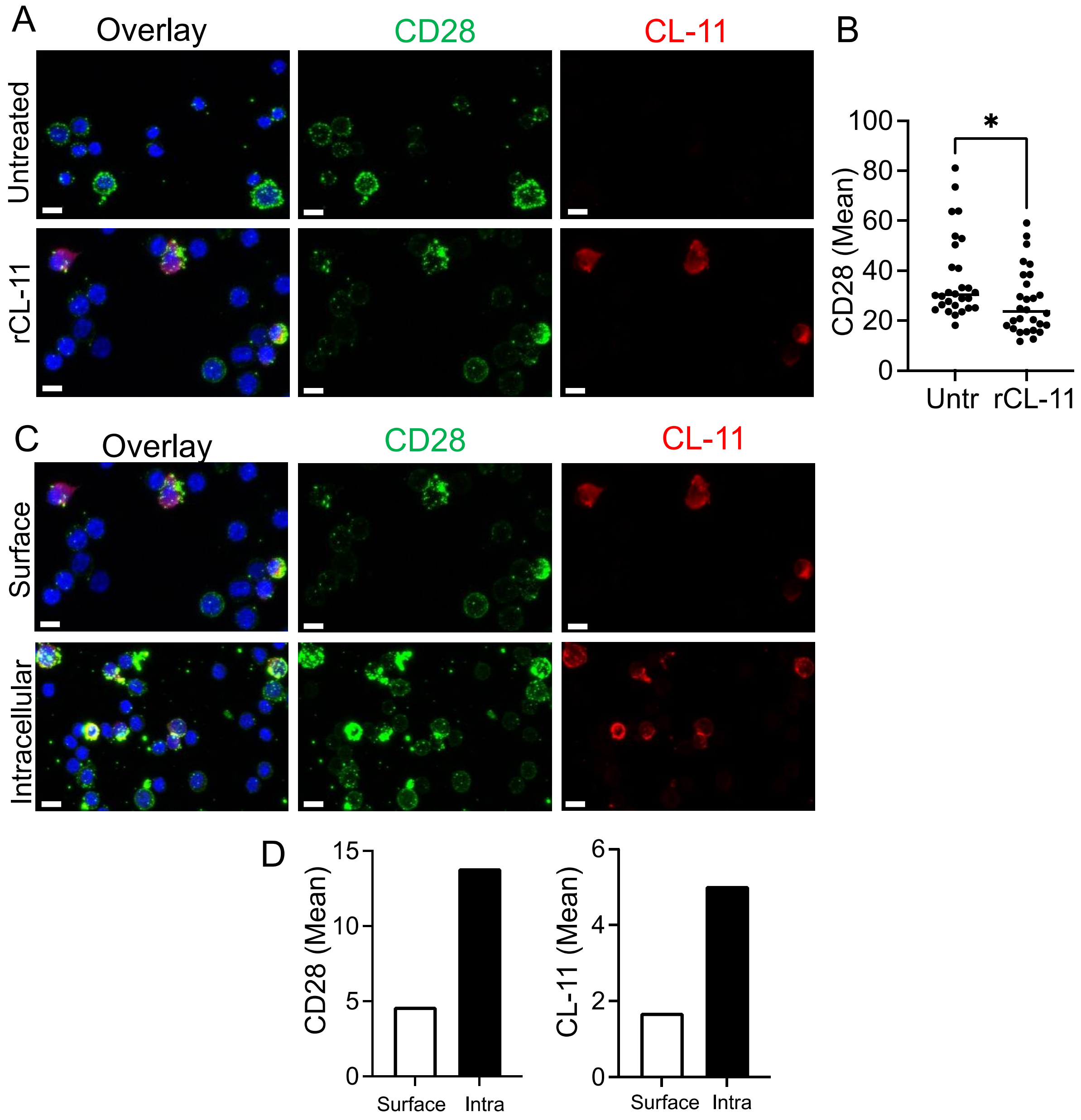

Figure S4. Surface versus Intracellular staining of CD28 and CL-11. (**A**) Representative high magnification images showing CD28 and CL-11 expression by non permeabilised $CD4^{+}CD25^{-}$ T cells activated with αCD3/αCD28 for 48 hours and pre-incubated for 1h at RT with buffer solution (untreated) or rCL-11. CD28 (green), CL-11 (red) and nuclei (blue) are shown. Scale bars represent 25 µM. (**B**) Quantification of CD28 mean

fluorescence using ImageJ software. Data are expressed as individual values (28) per condition (Untreated and rCL-11) *P<0.05 was considered significant using Two Tail Unpaired t test. (**C**) Representative high magnification cropped images showing CD28 and CL-11 expression by non permeabilised (Upper panels, Surface) and permeabilised (Bottom panels, Intracellular) $CD4^{+}CD25^{-}$ T cells activated with αCD3/αCD28 for 48 hours and pre-incubated for 1h at RT with rCL-11. CD28 (green), CL-11 (red) and nuclei (blue) are shown. Scale bars represent 25 μM. (**D**) Representative graph showing the CD28 and CL-11 mean fluorescence on non permeabilised (surface) and permeabilised (Intracellular) $CD4^{+}CD25^{-}$ T cells activated as described above.

Supplementary Table S1

Marker	Clone	Flurochrome	Supplier	Cat. No.
CD4	OKT4	Brillant Violet 605	BioLegend	317438
CD25	BC96	PE	BioLegend	302606
CD25	BC96	Brillant Violet 510	BioLegend	302640
CD3	UCHT1	Alexa Fluor 700	BioLegend	300424
CD8	SK1	PerCP-Cy5.5	BD Bioscience	565310
CD19	HIB19	Brilliant Violet 650	BioLegend	302237
CD28	CD28.2	APC	BioLegend	302912
HLA-DR	L243	Brilliant Violet 421	BioLegend	307636
HLA-DR	LN3	PE-Cy7	eBioscience	25-9965-42
PD-1	EH12.2H7	Brilliant Violet 785	BioLegend	329930
HLA-ABC	W6/32	APC	eBioscience	17-9983-42
HLA-ABC	W6/32	PerCP-Cy5.5	BioLegend	311420
CD80	2D10	Brilliant Violet 650	BioLegend	305225
CD86	2311(FUN-1)	Alexa Fluor 700	BD Bioscience	561124
PD-L1	MIH3	Brilliant Violet 421	BioLegend	374508
CD140b	18A.2	PE	BioLegend	323606
IL-10	JES3-19F1	PE	BioLegend	506804
IFN γ	4S.B3	PE-Cy7	BioLegend	502528
IL-17	BL168	Brilliant Violet 421	BioLegend	512322
TNFα	Mab11	APC	BioLegend	502912
